# Effectiveness of 4-Factor Prothrombin Complex Concentrate with and without Vitamin K in Managing Warfarin-Associated Major Bleeding

**DOI:** 10.5152/eurasianjmed.2025.25710

**Published:** 2025-04-14

**Authors:** Şeyma Arzu Temür, Mustafa Selçuk Ayar, Yiğit Kurnaz, Fatih Çalışkan

**Affiliations:** 1 Department of Emergency Medicine, Ondokuz Mayıs University Faculty of Medicine, Samsun, Türkiye; 2 Clinic of Emergency Medicine, Terme State Hospital, Samsun, Türkiye

**Keywords:** Blood coagulation factors, hemorrhage, international normalized ratio, vitamin K, warfarin

## Abstract

**Background:**

Major bleeding is a common and severe complication associated with vitamin K antagonist use. Rapid reversal of anticoagulation is crucial in cases of acute bleeding. Prothrombin complex concentrates (PCC) have emerged as an effective option. This study examines the efficacy of 4-factor PCC (4F-PCC) alone and in combination with vitamin K for International Normalized Ratio (INR) control in patients with life-threatening bleeding due to warfarin.

**Methods:**

This retrospective cross-sectional study included 50 patients who presented with bleeding and coagulopathy to the Ondokuz Mayıs University Health Practice and Research Center Adult Emergency Department between January 1, 2022, and January 1, 2023. Patients were divided into 2 groups based on vitamin K administration within 24 hours: group 1 received only 4F-PCC, while group 2 received both 4F-PCC and vitamin K.

**Results:**

The median age of patients was 72 years, 56% were male. The most common indication for warfarin use was heart valve disease (54%). Gastrointestinal hemorrhage was the leading cause of bleeding (44%). Mortality was 26%, with all deaths occurring within 72 hours of admission. Group 2 had significantly lower INR levels at 24th-hour than group 1 (*P* = .048). No significant differences were found in INR levels between the 1st and 24th hours within either group (*P* > .05).

**Conclusion:**

The study demonstrates that the combination of 4F-PCC and vitamin K is more effective in controlling INR than 4F-PCC alone, though it does not significantly impact rebound INR increases. Further prospective, multicenter studies are needed to confirm these findings and explore long-term outcomes.

**Main Points**

- The use of 4-factor prothrombin complex concentrates (4F-PCC) with vitamin K demonstrates superior international normalized ratio (INR) control within the first 24 hours compared to 4F-PCC alone in patients with warfarin-associated major bleeding.
- The combination of 4F-PCC and vitamin K does not show significant superiority over 4F-PCC alone in preventing rebound INR elevation, indicating a limited effect of vitamin K on rebound control.
- In emergency settings, 4F-PCC administration for warfarin-related major bleeding offers rapid INR reduction, providing effective hemostatic intervention within the critical first hours.
- Gastrointestinal bleeding is identified as the most common indication for 4F-PCC administration, underscoring its potential value in managing this patient group.

## Introduction

Vitamin K antagonists (VKAs) are orally available agents that prevent the gamma-carboxylation of vitamin K-dependent coagulation factors II, VII, IX, and X, converting them into functionally inactive proteins. They are widely used in the prophylaxis and treatment of venous thrombosis and pulmonary embolism.^1^ Bleeding is the most common complication encountered during the use of these medications.^2^ In cases of acute bleeding in patients using warfarin, the anticoagulation effect should be reversed before emergency surgical or interventional procedures.^3^ Traditionally, vitamin K and fresh frozen plasma have been used for this purpose, and in recent years, prothrombin complex concentrates (PCCs) have been included among the treatment options. Prothrombin complex concentrates are vitamin K-dependent factor concentrates with concentrations 25 times higher than those in plasma, allowing administration in a small volume and promoting factor regeneration and a decrease in international normalized ratio (INR) following administration. Current guidelines recommend 4-factor prothrombin complex concentrates (4F-PCC) for the reversal of VKAs.^4^ The effect of 4F-PCC and vitamin K administration on rebound INR elevation and its impact on INR control in patients with life-threatening bleeding due to warfarin use was evaluated in this study.

## Materials and Methods

### Patient Selection

This retrospective cross-sectional study included 50 patients who were admitted to the Ondokuz Mayıs University Health Practice and Research Center Adult Emergency Department with bleeding and coagulopathy between January 1, 2022, and January 1, 2023, and who were using warfarin within the indicated guidelines. Ethical approval for the study was obtained from the Ondokuz Mayıs University Clinical Research Ethics Committee (Approval no: 2024/200 Date: 15/05/2024), and the study was conducted in accordance with the Declaration of Helsinki. Informed consent was obtained from all patients for the anonymous use of their medical data for research purposes. For patients unable to provide consent due to critical medical conditions, consent was obtained from their legal guardians or authorized representatives. The collected data were fully anonymized, ensuring that no identifiable personal information was used in the study.

Patients eligible for inclusion were those with known warfarin use, aged over 18 years, presenting with coagulopathy (DIC), life-threatening bleeding (e.g., intracranial hemorrhage, gastrointestinal bleeding, intra-abdominal bleeding, or hematuria), and an INR value >1.5 with an indication for emergency surgery. Patients under 18 years of age and those who received fresh frozen plasma transfusion before 4F-PCC administration were excluded from the study.

The patients were divided into 2 groups based on the administration of vitamin K within the first 24 hours: group 1 consisted of patients who received only 4F-PCC within the first 24 hours, while group 2 included patients who received both 4F-PCC and vitamin K within the same period. Although vitamin K was intended to be administered to all patients, issues with drug supply prevented some patients from receiving it within the first 24 hours, influencing the group assignments. 4-Factor prothrombin complex concentrate (Cofact®) was administered according to the indication at the dose and duration specified in the weight-dose chart.

Data collected included age, gender, indication for warfarin use, presenting complaints, vital signs, INR values at admission, and at the 1st and 24th hour, indications for 4F-PCC use, vitamin K doses administered, and patient outcomes, all of which were recorded on the study form.

### Statistical Analysis

Data analysis was performed using IBM SPSS version 21.0 (IBM SPSS Corp.; Armonk, NY, USA). Frequencies were presented as the number (n) and percentage (%). The normality of the data was assessed using the Shapiro–Wilk test. Data that did not conform to a normal distribution were presented as the median (minimum-maximum). Repeated measurements of parameters within the same group were analyzed using the Friedman test. Differences between groups were evaluated using the Mann–Whitney *U* test. For all statistical analyses, a *P*-value of less than .05 was considered significant.

## Results

This cross-sectional retrospective study included 50 patients who presented to the emergency department with bleeding and met the inclusion criteria. The median age of the patients was 72 (22-88) years, and 56% (n = 28) were male. The most common indication for warfarin use was valvular heart disease, observed in 54% (n = 27) of the patients. Gastrointestinal hemorrhage was the most frequent complication associated with warfarin use and the primary indication for 4F-PCC administration, occurring in 44% (n = 22) of cases. Bleeding or bleeding-related complications resulted in death in 26% (n = 13) of the patients, all of whom died within 72 hours. Demographic data, indications for anticoagulant use, indications for 4F-PCC, and clinical outcomes are presented in Table 1.

The INR values at admission, 1st and 24th hour post-admission were compared between the groups. There was no statistically significant difference in INR values at admission and 1st-hour between the groups. However, the comparison of INR values at the 24th-hour demonstrated a statistically significant difference between the groups (*P* = .048) (Table 2).

When the INR values at admission, the 1st-hour, and the 24th-hour were analyzed within each group, a statistically significant difference was observed for both groups. This statistical difference was attributed to the INR values at admission for both groups (*P* < .001). However, no statistically significant difference was found between the 1st-hour and 24th-hour INR values within the groups (Table 3).

The significant change in INR values at admission and 1-24 hours reveals the efficacy of the treatment and the importance of early intervention in the emergency department.

## Discussion

Anticoagulants are used to prevent and treat thrombotic events associated with high morbidity and mortality, such as atrial fibrillation, heart valve replacement, stroke, and venous thromboembolism. However, major bleeding related to VKA use is a serious complication that requires immediate intervention.^1^ In cases of acute bleeding, the effect of VKA should be reversed as soon as possible, and the use of 3F-PCC or 4F-PCC is recommended for patients with bleeding.^5^ Patients using warfarin who presented to the emergency department with life-threatening bleeding and were treated with 4F-PCC were analyzed in this study. Of the participants, 56% were male, and the most common cause of life-threatening bleeding was gastrointestinal bleeding (44%). Yasaka et al^6^ investigated the efficacy and safety of 4F-PCC in 1271 patients with life-threatening bleeding and found that the most common cause of bleeding was gastrointestinal hemorrhage, particularly in males. Additionally, their study found that the most common cause of life-threatening bleeding was intracranial bleeding (59%). Another study identified gastrointestinal bleeding (32%) and intracranial bleeding (32%) as the most common causes of bleeding related to VKA use.^7^ It is believed that the prominence of gastrointestinal bleeding as the major cause in the patients of this study may be due to the high number of patients with chronic liver disease and esophageal varices being followed up at the center where the study was conducted.

In a study by Sombat et al^8^ on complications in patients using warfarin, it was found that the most common indication for warfarin use was atrial fibrillation, accounting for 60.9%. The primary indication for warfarin therapy in the cohort was the presence of a prosthetic heart valve, which constituted 54.0% of all cases. Patients’ knowledge and compliance level regarding warfarin is a crucial factor in achieving the target INR value.^9^ It is believed that the lower rate of atrial fibrillation in the sample, compared to the aforementioned study, may be due to the increased use of new generation oral anticoagulants in patients with atrial fibrillation, driven by patients’ noncompliance with warfarin therapy.

The mortality rate among patients with major bleeding receiving VKA therapy was 26%, with all deaths occurring within 72 hours of presentation to the emergency department. In a study examining mortality in patients using new-generation oral anticoagulants and VKAs, mortality due to major bleeding was found to be 26.2%.^10^ The mortality rate in this study was similar to the aforementioned study.

In the literature, the administration of 4F-PCC and vitamin K is recommended for effective INR control in patients using warfarin who have life-threatening bleeding.^6^,^11^,^12^In this study, a significant difference was observed between the groups when the 24th-hour INR values were analyzed. It was found that the 24th-hour INR value was lower in patients who received both 4F-PCC and vitamin K compared to those who received only 4F-PCC. This suggests that the administration of vitamin K alongside 4F-PCC is more effective for INR control in patients using VKAs who experience major bleeding, as stated in the literature.

In a study by Sin et al,^13^ rebound INR elevation was found in 25% of patients who received 4F-PCC for the reversal of warfarin effects, with 57.1% of these patients having received only 4F-PCC without vitamin K administration. In this study, the 1st-hour INR value was significantly lower in both the 4F-PCC-only group and the 4F-PCC plus vitamin K group compared to the INR value at admission. However, when each group was evaluated within itself, no statistically significant difference was observed between the INR values obtained at the 1st and 24th hours. The lack of a statistically significant difference between the 1st and 24th-hour INR values in both groups suggests that vitamin K has no effect on rebound INR elevation in patients on warfarin undergoing 4F-PCC treatment for major bleeding.

There are studies in the literature indicating that rebound INR elevation may occur in patients treated with fresh frozen plasma.^14^Additionally, some studies suggest improving the administration of 4F-PCC and vitamin K for bleeding related to VKA use.^15^ Although there are studies in the literature on INR control following 4F-PCC administration in patients with bleeding due to VKA use, there appears to be a lack of sufficient research regarding rebound INR elevation. While 4F-PCC administration is effective in controlling INR during the acute phase, it is believed that further studies are needed to address the issue of rebound INR elevation. In this study, it was found that the combined administration of 4F-PCC and vitamin K was more effective for acute-phase INR control compared to 4F-PCC alone in patients presenting to the emergency department with life-threatening bleeding. However, it was observed no significant effect of combining 4F-PCC with vitamin K on rebound INR elevation compared to the use of 4F-PCC alone.

The rapid and effective reduction of INR within the first 24 hours following the administration of 4F-PCC, regardless of adjunctive vitamin K, underscores the crucial role of early intervention in the management of VKA-associated major bleeding. Previous studies have consistently demonstrated the superiority of 4F-PCC over plasma in achieving rapid hemostasis and INR normalization, thereby reducing the need for additional transfusions and improving patient outcomes.^11^,^16^^-^ ^18^

The use of 4F-PCC in conjunction with vitamin K is effective for INR control in patients with major bleeding associated with VKAs; however, it does not significantly prevent rebound increases in INR. Further prospective, multicenter studies are needed to confirm these findings and explore long-term outcomes.

This study has several limitations that should be acknowledged. First, as a retrospective, single-center analysis, the findings are inherently subject to selection and information biases, limiting the generalizability of the results to broader populations. Second, the study design only allowed us to assess INR values up to 24 hours after treatment. Future studies with extended follow-up periods are necessary to address these aspects comprehensively.

Additionally, the study relied on medical records, which may lack granularity or completeness in reporting certain clinical parameters, such as the precise timing of vitamin K administration relative to 4F-PCC infusion or other adjunctive therapies. Furthermore, while differences in INR reduction between the 2 groups were observed, other clinically relevant outcomes such as hemostasis efficacy, transfusion requirements, or survival rates were not analyzed in depth. Including these outcomes in future research would provide a more holistic understanding of treatment efficacy.

Finally, logistical challenges, such as variations in drug availability, led to the unequal distribution of patients between the study groups. This might have influenced the study’s outcomes and should be considered when interpreting the results. Future multicenter, prospective studies with standardized treatment protocols are needed to validate our findings and explore the long-term implications of these therapeutic strategies.

## Figures and Tables

**Table 1.** Demographic Data, Indications for Anticoagulant Use, Indications for 4F-PCC Administration, and Clinical Outcomes

	n	%
**Gender**		
Male	28	56
Female	22	44
**Indication for anticoagulation**		
Prosthetic heart valve	27	54
Atrial fibrillation/flutter	12	24
Cerebrovascular disease	6	12
Pulmonary thromboembolism	5	10
**4F-PCC indication**		
Gastrointestinal hemorrhage	22	44
Need for surgery	11	22
Intracranial hemorrhage	9	18
Hematuria	3	6
Disseminated intravascular coagulopathy	3	6
Haemoptysis	2	4
**Clinical outcome**		
Full recovery	37	74
Exitus	13	26
	Median (min-max)	
**Laboratory**		
WBC (10^3^/µL)	10.7 (2.8-28.3)	
Hb (g/dL)	7.9 (3.9-15.3)	
PLT (10^3^/µL)	219.5 (88-512)	
INR	10.8 (2-25)	

4F-PCC, 4-factor prothrombin complex concentrates; Hb, hemoglobin; INR, international normalized ratio; PLT, platelet count; WBC, white blood cell.

**Table 2.** Comparison of INR Values Between Group 1 and Group 2

		**Group 1 (n = 22)** **(Only PCC)**	**Group 2 (n = 28)** **(PCC + Vitamin K)**	**Test Statistic**	P*
	Time				
INR	Admission	6.0 (1.97-25.0)	14.5 (2.6-25.0)	397.5	.073
	1st-hour	1.7 (0.9-5.3)	1.5 (1.1-5.3)	240.0	.184
	24th-hour	2.4 (0.9-25.0)	1.6 (1.1-5.3)	207.0	**.048**

INR, international normalized ratio.

*Mann–Whitney *U* test, median (min-max).

**Table 3.** Comparison of INR Values Within Each Group Over Time

		**Group 1 (n = 22)** **(Only PCC)**	**Test Statistic**	P*	**Group 2 (n = 28)** **(PCC + Vitamin K)**	**Test Statistic**	P*
	Time						
INR	Admission	6.0 (2.0-25.0)^a^	28.54	**<.001**	14.5 (2.6-25.0)^a^	39.21	**<.001**
	1st-hour	1.7 (0.9-5.3)^b^			1.5 (1.1-5.3)^b^		
	24th-hour	2.4 (0.9-25.0)^b^			1.6 (1.1-5.3)^b^		

INR, international normalized ratio.

*Friedman test, median (min-max).

^a,b^There is no statistically significant difference between time periods with the same letter.

## Data Availability

The data that support the findings of this study are available upon request from the corresponding author.
